# Incidence and transmission associated with respiratory viruses in an acute care facility: An observational study

**DOI:** 10.1017/ice.2024.25

**Published:** 2024-06

**Authors:** Abby L. Valek, Vatsala Rangachar Srinivasa, Ashley M. Ayres, Steven Cheung, Lee H. Harrison, Graham M. Snyder

**Affiliations:** 1 Department of Infection Prevention and Control, UPMC Presbyterian, Pittsburgh, PA, USA; 2 Division of Infectious Diseases, Department of Medicine, University of Pittsburgh School of Medicine, Pittsburgh, PA, USA; 3 Microbial Genomic Epidemiology Laboratory, Center for Genomic Epidemiology, University of Pittsburgh, Pittsburgh, PA, USA; 4 Department of Epidemiology, School of Public Health, University of Pittsburgh, Pittsburgh, PA, USA.; 5 School of Public Health, University of Pittsburgh, Pittsburgh, PA, USA

## Abstract

We estimated the extent of respiratory virus transmission over three pre-COVID-19 seasons. Of 16,273 assays, 22.9% (3,726) detected ≥1 respiratory virus. The frequency of putatively hospital-acquired infection ranged from 6.9% (influenza A/B) to 24.7% (adenovirus). The 176 clusters were most commonly associated with rhinovirus/enterovirus (70) and influenza A/B (62).

## Background

While the rate of hospital-associated respiratory virus infections has been infrequently described, the transmission patterns of these viruses outside of isolated outbreaks is not well described.^1–5^ The aims of this study were to describe the extent of respiratory virus transmission in the acute care setting and characterize the virus-specific frequency and potential pathways of respiratory virus transmission.

## Methods

### Setting and patient population

This study took place during three respiratory virus seasons (October–September 2017–2018, 2018–2019, and 2019–2020 stopped after February 2020 due to the COVID-19 pandemic) at two acute care hospitals; the characteristics of the hospitals are provided in Supplemental Table 1. This study included all patients with one or more respiratory virus assays performed between 10/1/2017 and 2/28/2020. The project underwent formal review and was granted ethical approval as a quality improvement project by the UPMC Quality Review Committee.

During the study period, ordering of respiratory virus testing was based solely on providers’ discretion. Patients with any respiratory virus test ordered (pending collection or result) are automatically ordered droplet and contact transmission-based precautions until the test is resulted; precautions used for a positive test are virus-dependent (Supplemental Table 2).

Test assays included a respiratory viral panel (RVP) (GenMark Diagnostics [Carlsbad, CA]) and influenza and RSV-only PCR testing (Rapid Flu/RSV, Cepheid [Sunnyvale, CA]). The RVP assay includes tests for influenza (A and B), parainfluenza (types 1, 2, 3, and 4), rhinovirus/enterovirus, adenovirus, coronavirus (not including SARS-CoV-1, SARS-CoV-2, or MERS-CoV), human metapneumovirus (HMPV), respiratory syncytial virus (RSV A and B), and *Mycoplasma pneumoniae*. Viruses with subtypes were combined into one category. *Mycoplasma pneumoniae* results were omitted from the analysis.

### Study design and statistical analysis

This observational study described the frequency and proportion of potentially hospital-acquired respiratory virus infections. Respiratory virus transmission was characterized in two ways: (1) quantifying the proportion of positive assays and tests defined as putatively community- or hospital-acquired among all positive assays and tests, respectively, and (2) characterizing clusters of respiratory virus transmission using spatiotemporal association. In the characterization of tests, hospital-acquired was defined as a positive test performed on or after hospital day 4 of the current encounter. All others were considered community-acquired. Readmissions were considered new hospital encounters.

The cluster analysis was performed using the first instance of a positive test for each virus for each patient per season during the study period. Patients could be included in the cluster analysis data multiple times if they had an assay which was positive for multiple viruses. However, patients who tested positive for the same virus more than once per season were excluded after the first positive test. A cluster was defined as 4 or fewer days in between at least 2 patients’ positive respiratory virus tests for the same virus on the same unit where there were at least 10 licensed inpatient beds. A patient’s location and attribution of the virus was based on the location where the respiratory virus test was collected, though it is possible that patients could be transferred throughout the hospital during their encounter. The cut off of 4 days was based on the median incubation period for respiratory viruses previously described.^6^ We also conducted a sensitivity analysis using either 2 or 7 days between patient test positivity dates as the cutoffs to define a potential cluster.

Unit location was used as a surrogate for immune status in this study. Patients with a positive respiratory virus test while admitted to a unit designated for care of immunocompromised individuals were denoted as such. These units, defined by the National Healthcare Safety Network (NHSN) using the 80/20 rule, included transplant ICUs, transplant stepdown floors, oncology ICUs, and oncology stepdown floors (https://www.cdc.gov/nhsn/pdfs/psc/mappingpatientcarelocations.pdf). These units implement special precautions for immune suppressed patients including droplet precautions for patients with solid organ transplants during influenza season, which is defined at our facility using the following criteria: (1) influenza-like-illness activity level for the state reaches “moderate” or greater (defined by CDC) and (2) influenza activity level for the state reaches “local activity” or greater (defined by the Pennsylvania Department of Health).

The statistical software R was used to analyze the data to identify clusters of infections based on overlapping time (date of respiratory virus testing) and space (hospital unit location) (https://www.R-project.org/).

## Results

A total of 16,273 respiratory virus specimens were collected between the two hospitals during the three-season study period, including 16,000 RVP assays and 273 (1.7%) influenza/RSV rapid PCR assays. The assay positivity frequency (for ≥1 virus) ranged from 20.9% to 28.1% for each hospital season (Supplemental Table 3) Seasonal trends in the number assays performed, number of assays positive for ≥1 pathogen, and the frequency of positive tests by viral pathogen are shown Supplemental Figures 1 and 2. Rhinovirus/enterovirus were the most frequently identified viruses in both community- and hospital-acquired cases, whereas the virus with the greatest percentage of cases classified as hospital-acquired (24.7%) was adenovirus (Table 1).

**Table 1.** All assays/tests analysis: Proportion of tests attributed to community versus hospital acquisition among all positive respiratory virus tests

Respiratory virus	Positive tests	Community-acquired (%)	Hospital-acquired (%)
Rhinovirus/Enterovirus	1,386	1,200 (86.6)	186 (13.4)
Influenza virus A and B	1,035	964 (93.1)	71 (6.9)
Respiratory syncytial virus	399	356 (89.2)	43 (10.8)
Coronavirus	330	280 (84.8)	50 (15.2)
Parainfluenza virus	306	266 (86.9)	40 (13.1)
Human metapneumovirus	285	257 (90.2)	28 (9.8)
Adenovirus	89	67 (75.3)	22 (24.7)

During the study period, there were a total of 176 respiratory virus clusters, ranging from 14 to 44 clusters per season in the two study hospitals (Table 2). Cluster sizes ranged from two to eight cases, with a median of two cases. In the sensitivity analysis adjusting the definition of temporal association, the total number of clusters may be 25% greater (220 clusters using 7-day cutoff) or 32% less (120 clusters using 2-day cutoff) than the base estimate (Supplemental Table 4). In the primary analysis, rhinovirus/enterovirus accounted for 39.8% (70/176) of clusters and influenza A and B viruses accounted for 35.2% (62/176) of clusters, and these two pathogens contributed the largest number of clusters in individual seasons and hospitals and in the sensitivity analyses. Among all patients with a positive respiratory virus test (N = 2,240), 17.9% (N = 400) were associated with a cluster; patients with influenza were most likely to be associated with a cluster (32.0%) (Supplemental Table 5). Immunocompromised units accounted for 19.9% (35/176) of clusters, predominantly due to rhinovirus/enterovirus infections (Supplemental Table 6).

**Table 2.** Cluster analysis: Number of potential clusters of respiratory virus transmission, by virus, season, and acute care facility

Virus	Hospital A			Hospital B			Hospital A and B
	2017–2018	2018–2019	2019–2020	2017–2018	2018–2019	2019–2020	All seasons
Rhinovirus/Enterovirus	14	14	4	13	17	8	70
Influenza virus A and B	14	9	8	20	7	4	62
Respiratory syncytial virus	3	3	1	3	4	2	16
Coronavirus	1	3	1	3	4	2	14
Parainfluenza virus	2	0	0	5	2	1	10
Human metapneumovirus	1	2	0	0	0	1	4
Adenovirus	0	0	0	0	0	0	0
Total	35	31	14	44	34	18	176

## Discussion

In this observational study of 3,726 positive respiratory virus assays among 2,240 patients over three respiratory virus seasons at two hospitals, we found approximately 7%–25% of positive tests to be presumptively hospital-acquired, and 0%–32% of patients with a positive test potentially associated with a cluster based on spatiotemporal analysis.

Rhinovirus/enterovirus and influenza accounted for 75% (132 of 176) of clusters, 32% of patients who tested positive for influenza were involved in a clustering event, and 20% (35/176) of clusters were attributed to units caring for most immunocompromised patients at the two hospitals. While we did not systematically analyze epidemiologic investigations in this analysis, a substantial proportion of these were not identified through routine infection prevention and control surveillance. Both influenza and rhinovirus potentially affect patients who are immunocompromised, elderly, or have other underlying lung conditions.^7–9^ These viruses, and units caring for immunologically vulnerable patients, should be the focus of transmission prevention efforts. Computer automation to ascertain clusters in real time may improve detection during respiratory virus seasons.

Limitations may include: generalizability of findings from two hospitals; potential mis-attribution of unit location of acquisition; and inference of nosocomial transmission based on admission date of testing and spatiotemporal associations. Subsequent studies should confirm or refute this method for assessing epidemiologic links between patients using genetic relatedness testing.^10^

Our analysis quantifies both the frequency of hospital-acquired respiratory viruses and a preliminary evaluation of transmission patterns. More detailed elucidation of influenza and rhinovirus/enterovirus transmission, particularly among immunocompromised patients, will help define improved transmission prevention measures.

## Supporting information

Valek et al. supplementary material Valek et al. supplementary material

## Supplementary material

The supplementary material for this article can be found at https://doi.org/10.1017/ice.2024.25.

## References

[1] Walker E , Ison MG. Respiratory viral infections among hospitalized adults: experience of a single tertiary healthcare hospital. Influenza Other Respir Viruses 2014;8:282–292. 24490751 10.1111/irv.12237 PMC4181476

[2] Huzly D , Kurz S , Ebner W , Dettenkofer M , Panning M. Characterisation of nosocomial and community-acquired influenza in a large university hospital during two consecutive influenza seasons. J Clin Virol 2015;73:47–51. 26540462 10.1016/j.jcv.2015.10.016 PMC7185613

[3] Chow EJ , Mermel LA. Hospital-acquired respiratory viral infections: incidence, morbidity, and mortality in pediatric and adult patients. Open Forum Infect Dis 2017;4:ofx006. 28480279 10.1093/ofid/ofx006 PMC5414085

[4] Manchal N , Mohamed MRS , Ting M , et al. Hospital acquired viral respiratory tract infections: an underrecognized nosocomial infection. Infect Dis Health 2020;25:175–180. 32205064 10.1016/j.idh.2020.02.002

[5] Petrie JG , Talbot TR. Health care-acquired viral respiratory diseases. Infect Dis Clin North Am 2021;35:1055–1075. 34752220 10.1016/j.idc.2021.07.007 PMC9759602

[6] Lessler J , Reich NG , Brookmeyer R , Perl TM , Nelson KE , Cummings DA. Incubation periods of acute respiratory viral infections: a systematic review. Lancet Infect Dis 2009;9:291–300. 19393959 10.1016/S1473-3099(09)70069-6 PMC4327893

[7] Kraft CS , Jacob JT , Sears MH , Burd EM , Caliendo AM , Lyon GM. Severity of human rhinovirus infection in immunocompromised adults is similar to that of 2009 H1N1 influenza. J Clin Microbiol 2012;50:1061–1063. 22205807 10.1128/JCM.06579-11 PMC3295181

[8] de Lima CR , Mirandolli TB , Carneiro LC , et al. Prolonged respiratory viral shedding in transplant patients. Transpl Infect Dis 2014;16:165–169. 24289829 10.1111/tid.12167 PMC7169780

[9] Lehners N , Tabatabai J , Prifert C , et al. Long-term shedding of influenza virus, parainfluenza virus, respiratory syncytial virus and nosocomial epidemiology in patients with hematological disorders. PLoS One 2016;11:e0148258. 26866481 10.1371/journal.pone.0148258 PMC4750950

[10] Sundermann AJ , Chen J , Miller JK , et al. Whole-genome sequencing surveillance and machine learning for healthcare outbreak detection and investigation: a systematic review and summary. Antimicrob Steward Healthc Epidemiol 2022;2:e91. 36483409 10.1017/ash.2021.241 PMC9726481

