# Supplementary material for: Incidence and transmission associated with respiratory viruses in an acute care facility: An observational study
Source: Infect Control Hosp Epidemiol. 2024 Feb 14;45(6):774–6. doi: 10.1017/ice.2024.25 (PMC11102818; doi:10.1017/ice.2024.25)
Supplement: Valek et al. supplementary material [file S0899823X24000254sup001.pdf]

SUPPLEMENTAL MATERIALS

Table of Contents

Page 2	SUPPLEMENTAL TABLE 1. Characteristics of study hospitals
Page 3	SUPPLEMENTAL TABLE 2. Transmission-based isolation precautions and personal protective equipment required by virus type
Page 4	SUPPLEMENTAL TABLE 3. All assays/tests analysis: Respiratory virus assays performed at the two study acute care facilities during three respiratory virus seasons
Page 5	SUPPLEMENTAL FIGURE 1. All assays/tests analysis: Positive and negative respiratory virus assays over time
Page 6	SUPPLEMENTAL FIGURE 2. All assays/tests analysis: Positive respiratory virus assay results over time, by virus
Page 7	SUPPLEMENTAL TABLE 4. Cluster analysis: Sensitivity analysis of cluster frequency varying definition of temporal association
Page 8	SUPPLEMENTAL TABLE 5. Cluster analysis: Patients with a positive respiratory virus test and proportion involved in a cluster, by virus and hospital
Page 9	SUPPLEMENTAL TABLE 6. Cluster analysis: Respiratory virus clusters in immunocompromised patients, by location

SUPPLEMENTAL TABLE 1. Characteristics of study hospitals

**Characteristic**	**Hospital A**	**Hospital B**
Specialty area	Solid organ transplant	Hematology/oncology
Staffed inpatient beds, total	695	520
Intensive care unit beds (percent of total)	134 (19%)	66 (13%)
Non-intensive care unit beds (percent of total)	561 (81%)	454 (87%)
Proportion of non-intensive care unit beds in double-bedded rooms, percent	54%	22%
Total number of hospital units	33	20
Intensive care units, number (percent of total)	9 (27%)	5 (25%)
Non-intensive care units, number (percent of total)	24 (73%)	15 (75%)
Units for care of immunocompromised patients, number (percent of total)*	7 (21%)	4 (20%)

** Includes both intensive care and non-intensive care units.*

SUPPLEMENTAL TABLE 2. Transmission-based isolation precautions and personal protective equipment required by virus type

**Virus**	**Transmission-based Precautions**	**Required Personal Protective Equipment (PPE)**
Rhinovirus/Enterovirus	Droplet	Surgical mask
Influenza virus A and B		
Respiratory syncytial virus	Contact + Standard	Gown and gloves*
Human metapneumovirus		
Parainfluenza virus		
Adenovirus	Droplet and Contact	Surgical mask<br>Gown and gloves
Coronavirus		

SUPPLEMENTAL TABLE 3. All assays/tests analysis: Respiratory virus assays performed at the two study acute care facilities during three respiratory virus seasons

	**Hospital A**			**Hospital B**			**Hospital A & B**		
**Respiratory virus season**	Number of assays performed	Positive for ≥1 pathogen	Negative for all pathogens	Number of assays performed	Positive for ≥1 pathogen	Negative for all pathogens	Number of assays performed	Positive for ≥1 pathogen	Negative for all pathogens
2017-2018	3104	656 (21.1%)	2448 (78.9%)	2839	798 (28.1%)	2041 (71.9%)	5943	1454 (24.5%)	4489 (75.5%)
2018-2019	3343	713 (21.3%)	2630 (78.7%)	3198	762 (23.8%)	2436 (76.2%)	6541	1475 (22.6%)	5066 (77.4%)
*2019-2020**	1825	387 (21.2%)	1438 (78.8%)	1964	410 (20.9%)	1554 (79.1%)	3789	797 (21.0%)	2992 (79.0%)
Combined seasons	8272	1756 (21.2%)	6516 (78.8%)	8001	1970 (24.6%)	6031 (75.4%)	16273	3726 (22.9%)	12547 (77.1%)

SUPPLEMENTAL FIGURE 1. The number of respiratory virus assays over time at two study hospitals, including assays positive for one or more pathogens ("positive") and tests negative for all pathogens in the assay ("negative")

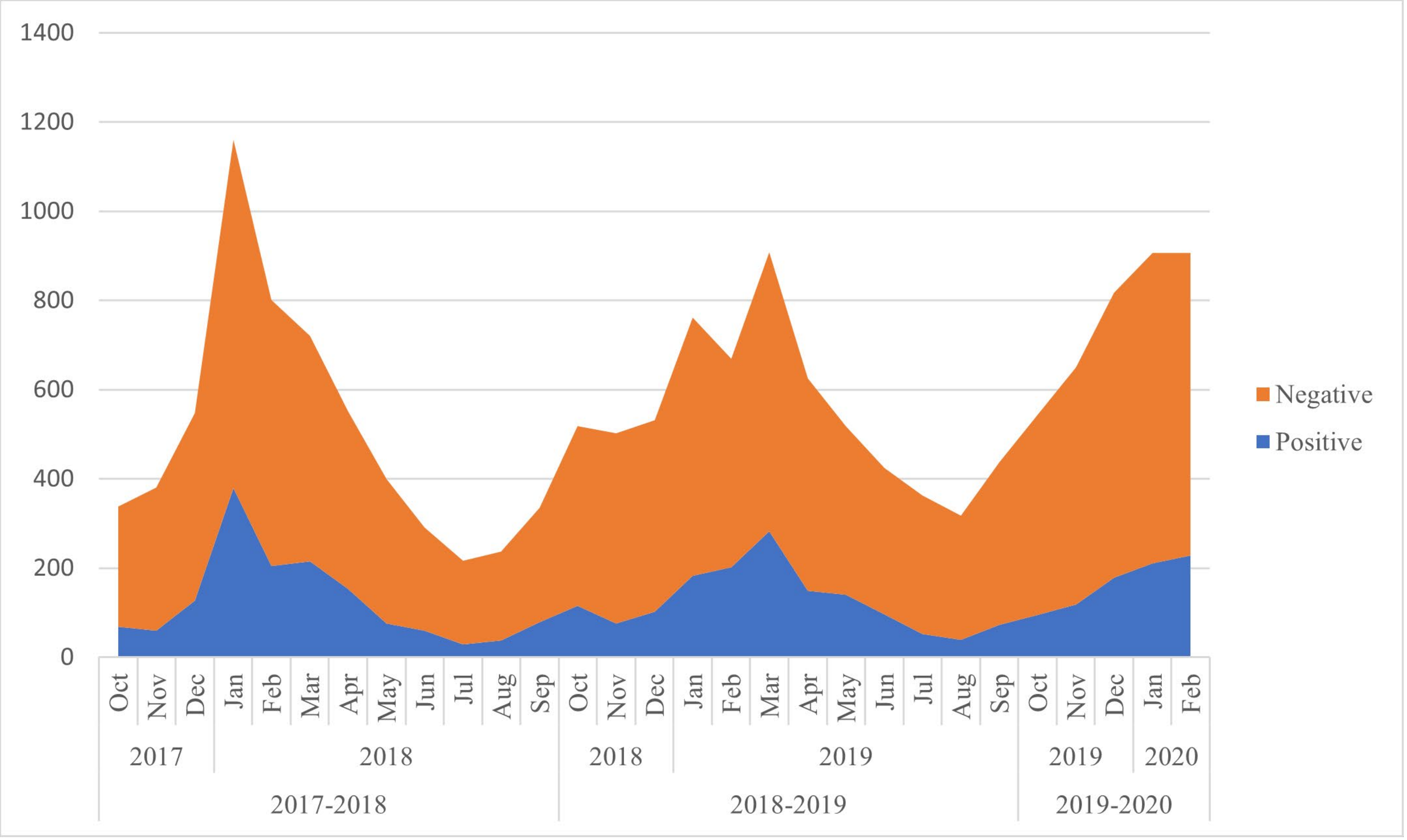

SUPPLEMENTAL FIGURE 2. Positive respiratory virus assay results over time, by virus

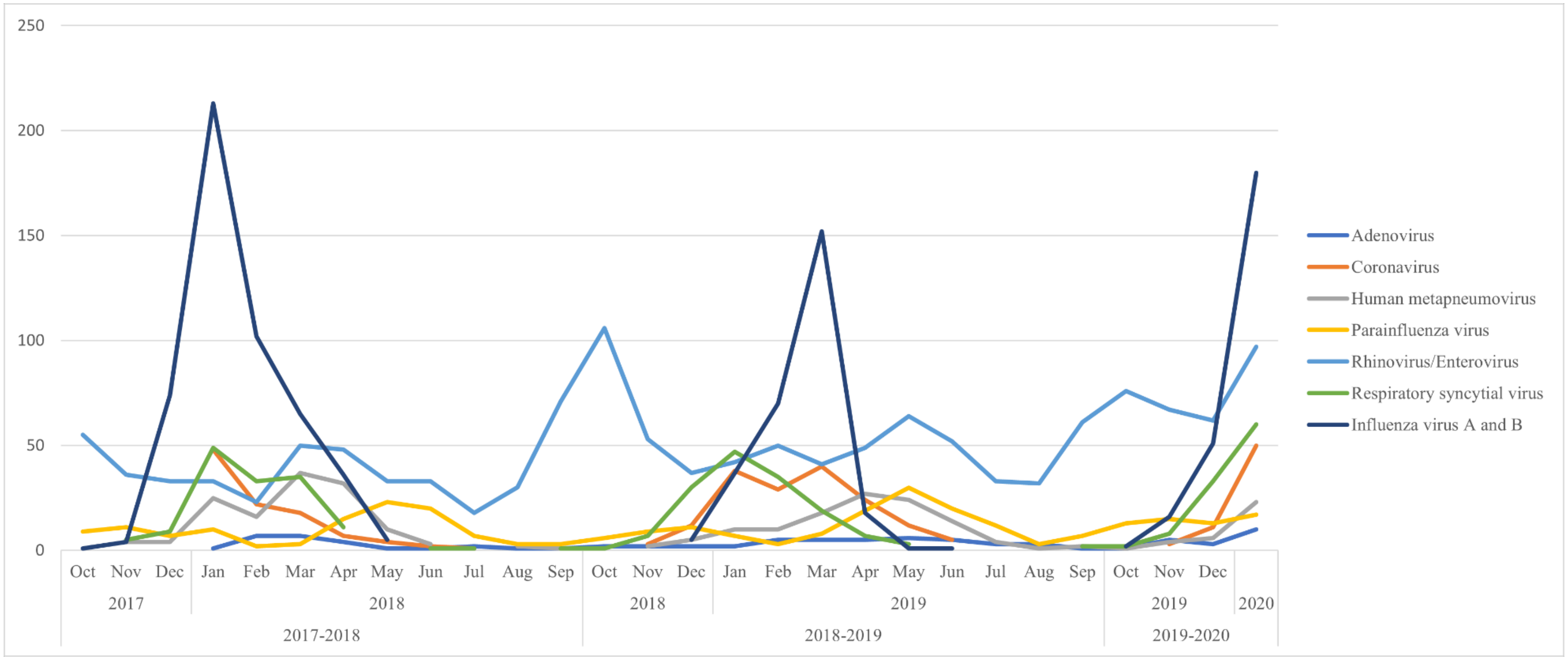

SUPPLEMENTAL TABLE 4. Sensitivity analysis of cluster frequency varying definition of temporal association

**Virus**	**Number of clusters using varying criteria for temporal association**					
	7-day cutoff (%)		4-day cutoff (%)		2-day cutoff (%)	
Rhinovirus/Enterovirus	102	46%	70	40%	45	38%
Influenza virus A and B	78	35%	62	35%	42	35%
Respiratory syncytial virus	13	6%	16	9%	5	4%
Coronavirus	15	7%	14	8%	15	13%
Parainfluenza virus	9	4%	10	6%	8	7%
Human metapneumovirus	3	1%	4	2%	5	4%
Adenovirus	0	0%	0	0%	0	0%
Total	220		176		120	

SUPPLEMENTAL TABLE 5. Cluster analysis: Patients with a positive respiratory virus test and proportion involved in a cluster, by virus and hospital

	**Hospital A**				**Hospital B**			
**Virus**	Patients with a positive test	Patients involved in a cluster (%)	Number of clusters	Median cluster size (range)	Patients with a positive test	Patients involved in a cluster (%)	Number of clusters	Median cluster size (range)
Rhinovirus/ Enterovirus	441	74 (16.8)	32	2 (2-4)	413	80 (19.4)	38	2 (2-3)
Influenza virus A & B	238	76 (31.9)	31	2 (2-8)	256	82 (32.0)	31	2 (2-7)
Respiratory syncytial virus	109	14 (12.8)	7	2*	129	19 (14.7)	9	2 (2-3)
Coronavirus	93	10 (10.8)	5	2*	118	17 (14.4)	9	2 (2-3)
Parainfluenza virus	100	4 (4.0)	2	2*	109	16 (14.7)	8	2*
Human metapneumovirus	82	6 (7.3)	3	2*	95	2 (2.1)	1	–
Adenovirus	32	0 (0)	0	–	25	0 (0)	0	–

* All clusters N=2

SUPPLEMENTAL TABLE 6. Respiratory virus clusters in immunocompromised patients, by location

	**Hospital A**		**Hospital B**	
**Virus**	Transplant ICU (2 units)	Transplant Stepdown (5 units)	Medical ICU (1 units)	Oncology Stepdown (3 units)
Rhinovirus/Enterovirus	1	3	1	15
Influenza virus A and B	3	2	0	2
Respiratory syncytial virus	0	2	0	3
Parainfluenza virus	0	0	0	2
Coronavirus	0	0	0	1
Human metapneumovirus	0	0	0	0
Total	4	7	1	23

Note: ICU, intensive care unit
